# Sloping Farmlands Conversion to Mixed Forest Improves Soil Carbon Pool on the Loess Plateau

**DOI:** 10.3390/ijerph19095157

**Published:** 2022-04-24

**Authors:** Binbin Li, Xuejian Shen, Yongjun Zhao, Peijuan Cong, Haiyan Wang, Aijuan Wang, Shengwei Chang

**Affiliations:** Water and Soil Conservation Monitoring Center of Ministry of Water Resources, Beijing 100055, China; sxj@mwr.gov.cn (X.S.); yongjun_zhao@163.com (Y.Z.); cpj1103@163.com (P.C.); whaiyan363@163.com (H.W.); wang-ai-juan@163.com (A.W.); csw200222000011@126.com (S.C.)

**Keywords:** afforestation, carbon sequestration, forest soils, land management, land use, soil organic carbon

## Abstract

Vegetation restoration is considered a potentially useful strategy for controlling soil erosion and improving soil organic carbon (SOC) in arid and semiarid ecosystems. However, there is still debate regarding which vegetation restoration type is the best choice. In this study, four vegetation restoration types (i.e., grasslands, shrubs, forests and mixed forests) converted from sloping farmlands were selected to explore the SOC variation among the four types and to investigate which soil factors had the greatest effect on SOC. The results showed while the magnitude of effect differed between vegetation restoration type, all studied systems significantly increased SOC and labile organic carbon contents (*p* < 0.01), soil nutrients such as total nitrogen (TN) (*p* < 0.01), available nitrogen (AN) (*p* < 0.01), total phosphorus (TP) (*p* < 0.05) and available phosphorus (AP) (*p* < 0.05), soil enzyme activities such as phosphatase (*p* < 0.01), soil microbial biomass carbon (MBC) (*p* < 0.05), and basal respiration (BR) (*p* < 0.05), but had significant negative correlationswith polyphenol oxidase (*p* < 0.05). However, the effects of vegetation restoration of farmland converted to natural grasslands, shrubs, forests and mixed forests varied. Among the types studied, the mixed forests had the largest overall positive effects on SOC overall, followed by the natural grasslands. Soil nutrients such as N and P and soil microbial activities were the main factors that affected SOC after vegetation restoration. Mixed forests such as *Robinia pseudoacacia* and *Caragana korshinskii* are the best choice for farmland conversion on the central of the Loess Plateau.

## 1. Introduction

Soils play an important role in the global carbon cycle [1,2,3]. Soil organic carbon (SOC) is an essential physical-chemical soil property and the most important indicator of soil quality [4]. Vegetation restoration is considered to be a potentially useful strategy for controlling soil erosion and improving soil quality in arid and semiarid ecosystems [5,6,7]. The conversion of farmland to forests or grasslands has been shown to increase SOC by increasing C derived from new vegetation, thus simultaneously decreasing C loss from decomposition and erosion [8,9]. Thus, afforestation and revegetation have been proposed as effective methods for reducing atmospheric CO_2_ due to C sequestration in soils.

Soil physical-chemical properties have been extensively used to evaluate SOC; however, these properties usually change slowly, and thoroughly reflecting soil changes through these properties is impossible; thus, the selection of indicators that appropriately reflect the overall change in SOC is important. Previous studies have mainly focused on SOC dynamics during vegetation restoration [5], the effects of land use change and SOC [10,11], and C-N relations [9], as well as the effects of aspect-vegetation complexes on the decomposition of SOC [12]. Moreover, many studies have focused on the effects of soil microbes [13], soil enzyme activities [14,15], soil nutrients [16], soil aggregates and SOC fractions [17,18], and soil mechanical components, e.g., sand, silt, and clay [19] on SOC. However, most of those studies only reported the effects of soil factors on SOC from one or a few aspects. For example, one study reported soil organic carbon variation determined by biogeographic patterns of microbial carbon and nutrient limitations [20], which can be reflected by the soil extracellular enzyme activities [6]. Soil enzyme-mediated mineralization of soil organic matter is a vital biochemical process within the soil C cycles [6]. And litter decomposition following vegetation restoration was linked to soil nutrient dynamics [21]. So, there is little available information on the combination of soil physical, chemical and biological factors to examine the effect of soil factors on SOC as a whole.

In 1999, the Chinese government implemented the “Grain for Green” Program (GGP) by restoring degraded farmland to forests, shrubs and grasslands [5]. Although the initial goal of the GGP program was aimed at controlling soil erosion and restoring ecosystems, it has been instrumental in increasing both the rate and overall quantity of C sequestered in the soil. At present, the ‘‘Grain for Green’’ program is the first and still the most ambitious, ecosystem services program in the world [22,23]. The Loess Plateau is the key zone for implementing the GGP. The process of natural and artificial restoration of abandoned farmland is underway on the Loess Plateau [5]. Although the initial goal of the GGP was to control soil erosion, the program strongly affects soil C cycling. Consequently, many studies have focused on changes in soil C accumulation following farmland conversion on the Loess Plateau [5,13,24]. However, those studies only focus on one simple type of vegetation restoration. The GPP includes forests, shrubs, grasslands and mixed forests. To date, there is still controversy regarding the best choice of vegetation restoration type for the Loess Plateau.

Therefore, we are in need of a comprehensive study of soil C variations that considers different types of vegetation restoration (i.e., forests, shrubs, grasslands and mixed forests). The objectives of the study were to (1) explore the difference in SOC under different vegetation restoration types and (2) identify the soil factors that have the greatest effect on SOC.

## 2. Materials and Methods

### 2.1. Study Area

**Figure 1:**
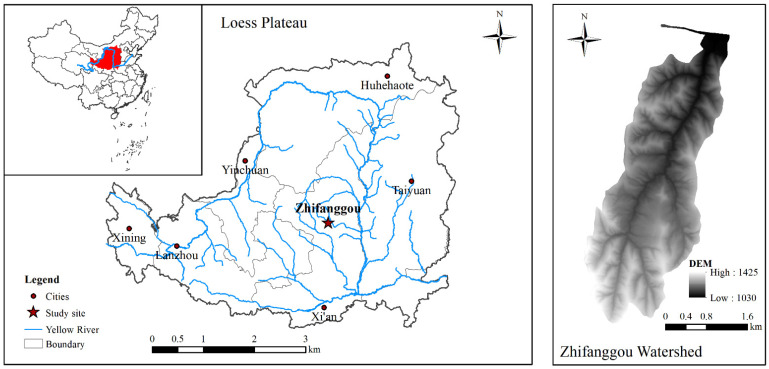
Location of the Zhifanggou watershed on the Loess Plateau, China.

This study was conducted in the Zhifanggou watershed in Ansai County, Shaanxi Province, NW China (36°46′28″–36°46′42″ N, 109°13′46″–109°16′03″ E; 1010–1400 m a.s.l., 8.27 km^2^) (Figure 1). The study area is characterized by a semiarid climate and a deeply incised hilly-gully loess landscape. Slopes vary between 0° and 65°. The Zhifanggou watershed is a popular case study area for comprehensive soil and water conservation on the Loess Plateau. The mean annual temperature range is 9.1 °C (from 1970 to 2010). The average maximum temperature is 36.8 °C and the average minimum temperature is −23.6 °C in the whole year; the average frost-free period is 157 days. The mean annual precipitation is 503 mm (from 1970 to 2010), of which 70% falls between July and September. Soil types are classified as a typical loess soil (*Calcic Cambisols*) and are susceptible to erosion. The main herbaceous plants are *Stipa bungeana*, *Bothriochloa ischaemum*, *Artemisia sacrorum*, *Potentilla acaulis*, *Stipa grandis*, *Androsace erecta*, *Heteropappus altaicus*, *Lespedeza bicolor*, *Artemisia capillaris* and *Artemisia frigid*, of which *S. bungeana* is the most widely distributed. In addition, shrubs such as *Rosa xanthina*, *Spiraea pubescens* and *Hippophae rhamnoides* can be found in gullies. The primary planted trees in the study area are *Robinia pseudoacacia*, *Populus simonii*, *Caragana microphylla* and *Platycladus orientalis* [13].

### 2.2. Experimental Design and Soil Sampling

Five land use types, sloping farmland (SL), grassland (GL, natural restoration), shrubland (SL, *Caragana korshinskii*), forestland (FL, *Robinia pseudoacacia*), and mixed forests (ML, *Robinia pseudoacacia + Caragana korshinskii*, in the watershed were selected for study. Three forest types were planted on sloped farmlands, and the grasslands developed from abandoned sloped farmlands (control). Between 1988 and 1990, all forests, shrubs and grasslands were planted or naturally restored by the local farmers. In 2018, our project team established 12 plots in these afforested systems. Management histories for the 30 years of plant growth were obtained by interviews with local farmers and village elders and by reviewing rental contracts between farmers and the government.

In each vegetation restoration type, three 20 m × 20 m plots were established in August 2018 when the plant biomass peaked. Five quadrats (1 m × 1 m) were separately chosen in each of the four corners and center of the plots. Litter horizons were removed before soil sampling. Soil sampling, using a soil drilling sampler (9 cm inner diameter), was performed in the 0–20 cm soil layers. We then mixed the same layers together to form one sample. All samples were sieved through a 2 mm screen, and roots and other debris were removed in the field. Each sample was air-dried and stored at room temperature for the determination of soil physical and chemical properties. The soil bulk density (g cm^−3^) of the different soil layers was measured using a soil bulk sampler with a 5 cm diameter and 5 cm high stainless steel cutting ring (3 replicates) at points adjacent to the soil sampling quadrats. The original volume of each soil core and its dry mass after oven-drying at 105 °C over 48 h were measured for bulk density determination. The morphological traits of the herbage in each age group are listed in Table 1. The plots were all located near the top of the loess mounds. All plots were located in the hill-slope, loess-derived soil and north-faced slope. And there was little difference among the sites in regard to gradient, altitude, or previous farming practices.

### 2.3. Laboratory Assay

Soil pH was determined at a soil/water ratio of 1:2.5 (PHSJ-4A pH meter, Zhangqiu Meihua International Trading Co., Jinan, China). Soil bulk density (BD) was determined using the ring cutting method [13]. SOC was assayed by dichromate oxidation [25], and total nitrogen (TN) was assayed using the Kjeldahl method [26]. The available nitrogen (AN) was determined by the continuous alkali-hydrolyzed reduction diffusion method [27]. The total P (TP) and available P (AP) were determined by the Olsen method [28]. The soil labile organic carbon content (LOC) was determined following the method of Vieira et al. [29], and soil non-labile organic carbon content (NLOC) was determined by the SOC minus the LOC [13]. The soil particle sizes (clay, silt and sand contents) were determined using the MasterSizer 2000 method (Malvern MasterSizer 2000, Worcestershire, UK). Enzyme activities were assayed according to colorimetric determination methods [30,31]. All soil enzyme activities were determined using three replicates per sample. Microbial biomass C, N, and P contents (MBC, MBN, MBP, respectively) were analyzed by the chloroform fumigation-extraction method [20]. Soil basal respiration (BR) was estimated via CO_2_ evolution at 25.8 °C in samples incubated for 14 days, adjusted to 50% of the field water-holding capacity. The metabolic quotient (qCO_2_) was calculated as the ratio of soil basal respiration to microbial biomass C (BR/Cmic) [6,13].

### 2.4. Statistical Analysis

One-way ANOVA was used to analyze the means among different ecosystem types. Differences were evaluated at the 0.05 significance level. When significance was observed at the *p* < 0.05 level, Tukey’s post hoc test was used to carry out the multiple comparisons. Pearson correlation was used to indicate the relationships between SOC and the 23 other soil properties. Moreover, multivariable linear regression analysis (MLRA) was used to quantify the effects of soil factors on SOC. In the analysis, the absolute value coefficient was used as an indicator of the effect size and was summed to determine the relative contribution (RC) rates of soil and microbial properties in explaining the SOC. All analyses were performed using SPSS 25.0 (SPSS Inc., Chicago, IL, USA).

## 3. Results

### 3.1. SOC and Soil N and P Nutrients in Different Vegetation Restoration Types

**Figure 2:**
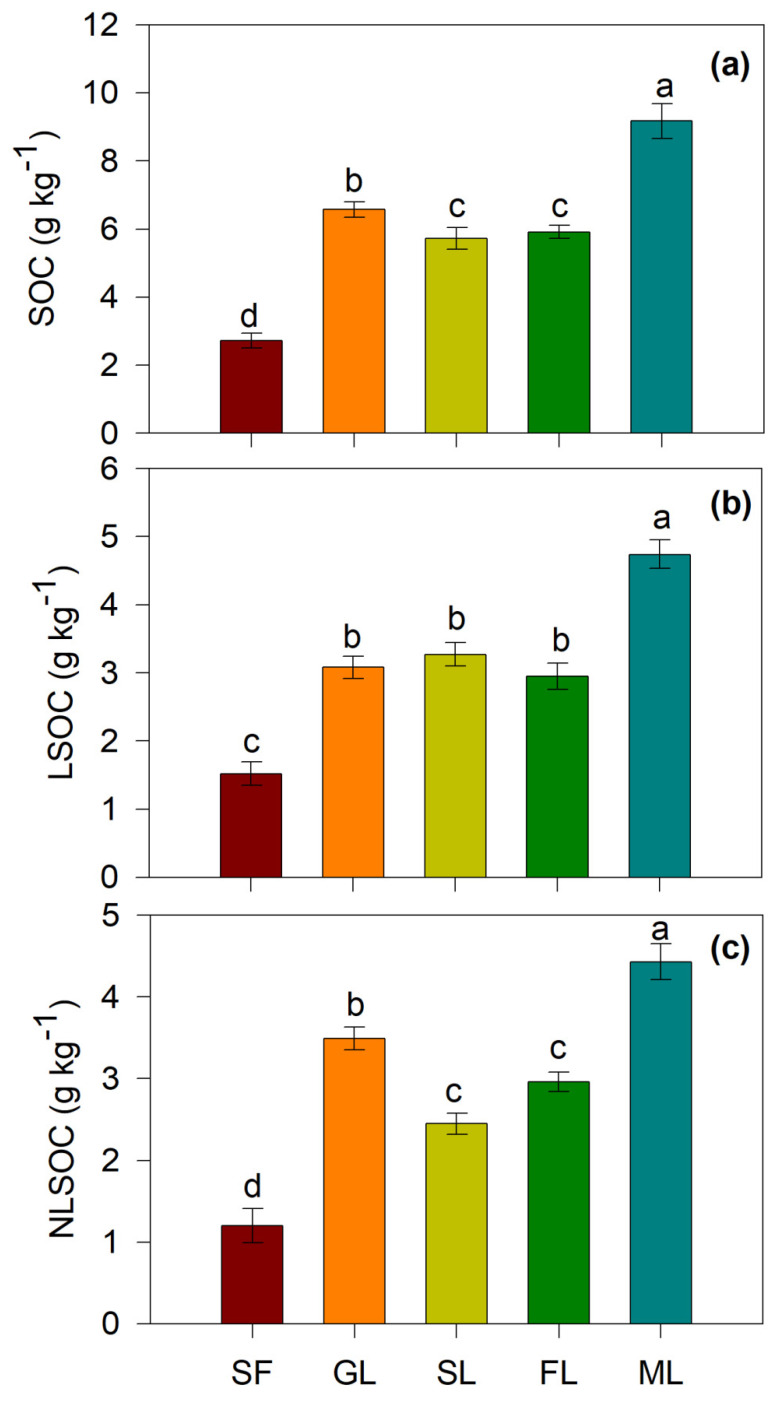
Soil organic carbon (SOC, (**a**)), soil labile organic carbon (LSOC, (**b**)) and Non-labile organic carbon (NLSOC, (**c**)) in different vegetation restoration types. Note: SF, Sloping farmland; GL, Grassland; SL, shrubland of *Caragana korshinskii*; FL, forestlands of *Robinia pseudoacacia*; ML, mixed forests of *Robinia pseudoacacia* + *Caragana korshinski*. Different lower-case letters above the error bars indicate significant differences in different land use types at 0.05 level (*p* < 0.05). Data are Means ± SE. *n* = 3.

**Figure 3:**
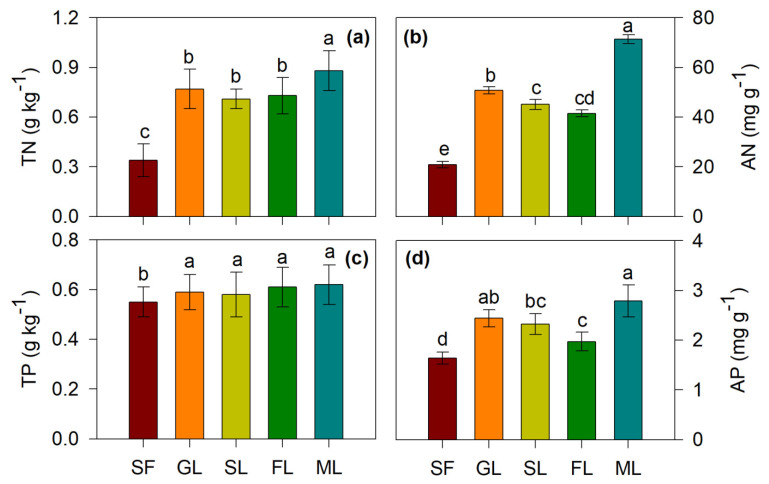
Soil N and P nutrients in different vegetation restoration types. TN (**a**), total nitrogen; AN (**b**), available nitrogen; TP (**c**), total phosphorus; AP (**d**), available phosphorus. Note: SF, Sloping farmland; GL, Grassland; SL, shrubland of *Caragana korshinskii*; FL, forestlands of *Robinia pseudoacacia*; ML, mixed forests of *Robinia pseudoacacia* + *Caragana korshinski*. Different lower-case letters above the error bars indicate significant differences in different land use types at 0.05 level (*p* < 0.05). Data are Means ± SE. *n* = 3.

All studied vegetation restoration types significantly increased SOC, LOC, NLOC, TN, TP, AN and AP compared with sloping farmland (*p* < 0.05) (Figure 2 and Figure 3). Among the four vegetation restoration types, i.e., grasslands, shrublands, forestlands and mixed forests, the mixed forests had the largest effect on SOC. The SOC in the mixed forests increased by 6.45 g kg^−1^ after 30 years of farmland conversion to mixed forests (Figure 2). In addition, vegetation restoration decreased soil pH and BD (Table 2). The soil silt and clay contents also increased overall after vegetation restoration (Table 1).

### 3.2. Soil Microbial Activities in Different Vegetation Restoration Types

All vegetation restoration had a significant effect on soil enzyme activities (Table 2). Following farmland abandonment, grassland exhibited decreased urease activities (*p* < 0.05), and shrubs, forests and the mixed forests exhibited increased urease activities (*p* < 0.05) (Table 2). Overall, amylase and polyphenol oxidase activities were reduced by vegetation restoration, but phosphatase, saccharase, cellulase, and catalase activities increased compared to sloping farmland (*p* < 0.05) (Table 2).

**Figure 4:**
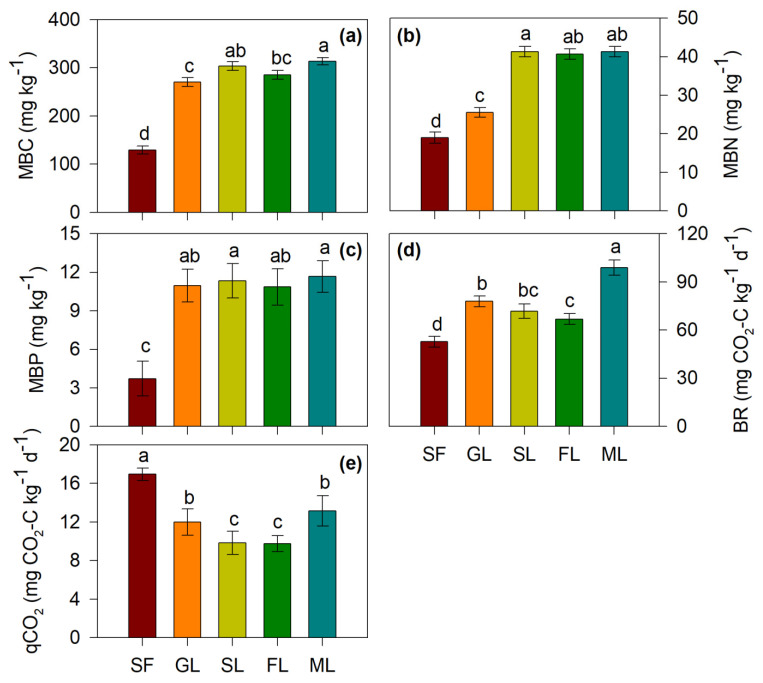
Microbial biomass C, N, P, respiration strength and qCO_2_ values of soils in the seven land use types. MBC (**a**), Microbial biomass carbon; MBN (**b**), Microbial biomass nitrogen; MBP (**c**), Microbial biomass phosphorus; BR (**d**), Basal respiration; qCO_2_ (**e**)_,_ Microbial respiratory quotient. Note: SF, Sloping farmland; GL, Grassland; SL, shrubland of *Caragana korshinskii*; FL, forestlands of *Robinia pseudoacacia*; ML, mixed forests of *Robinia pseudoacacia* + *Caragana korshinski*. Different lower-case letters above the error bars indicate significant differences in different land use types at 0.05 level (*p* < 0.05). Data are Means ± SE. *n* = 3.

All studied vegetation restoration types significantly increased MBC, MBN, and MBP contents compared with sloping farmland (*p* < 0.05) (Figure 4), suggesting that microbial biomass was increased due to vegetation restoration, which resulted in higher BR in grasslands, shrubs, forests and mixed forests (Figure 4). Among the types studied, the mixed forest had the highest MBC, MBN and MBP; the same result was observed for BR (Figure 4). However, the microbial respiratory quotient (qCO_2_) was reduced after farmland was converted to grasslands, shrubs, forests and mixed forests (Figure 4).

### 3.3. Factor Effects on SOC

SOC was related to soil nutrients, physical properties and microbial activities. Among the factors, SOC had a significant positive correlation with the TN, AN, TP and AP contents (*p* < 0.01) (Table 3). In addition, SOC also had a positive correlation with the phosphatase, saccharase, cellulase, and catalase activities, but it was negatively correlated with polyphenol oxidase activities (*p* < 0.05) (Table 3). SOC was also positively correlated with the MBC content and BR (*p* < 0.01) (Table 3). Based on the relative contribution (RC) analysis, the results showed that soil microbial activities contributed 45.1% to the SOC and that soil nutrients contributed 22%. All soil factors in the study contributed 87.7% to SOC in the multivariable linear regression analysis (Table 3).

## 4. Discussion

### 4.1. Vegetation Restoration Types Affect SOC and Soil N and P Properties

Land-use change following farmland conversion can cause a change in soil C [11]. The mixed forests had the largest positive effects on SOC, followed by the grasslands (Figure 2), because the mixed forests and natural grassland consume less soil water than single shrubs and trees in arid and semiarid regions [32], leading to a higher soil water content or unchanged soil water following vegetation restoration [32]. Higher soil moisture will promote plant growth and thus produce more plant biomass and litter input into the soils, consequently improving the accumulation of SOC [2]. In addition, the mixed forests had the largest positive effects on soil N and P compared with the sloping farmland (Figure 3). The direct possible reason is that mixed forests resulted in the greatest increase in SOM among the four vegetation restoration types in the study area. Usually, different vegetation types provide different surface residues and root distributions [33], leading to varied soil N content. For example, the patterns of soil N dynamics differed greatly among different tree species used in afforestation and depended on the transfer of soil organic matter (SOM) into soil via the roots of ground vegetation and litter decomposition [9]. Soils with different vegetation undergo different litter decomposition processes and rates, meaning that the release of N and P in soil differs [34]. Generally, all vegetation restoration types significantly increased TN, TP, AN and AP content (Figure 3), mainly because vegetation cover, plant species and biomass increased markedly after farmland abandonment [6]. Ground litter decomposition by microbes and root extension may contribute the most to soil N and P accumulation [34,35].

### 4.2. Effects of Vegetation Restoration Type on Soil Microbial Activities

Soil enzymatic activity plays an important role in C cycling and nutrient dynamics [36]. As sensitive indicators of the influence of land use changes or vegetation restoration on soil [6], changes in plant cover, SOC, and soil environmental conditions (e.g., pH and BD) after farmland abandonment would change the soil microbial composition and enzyme activity [6,36]. Plant residues in afforested ecosystems contain more roots and substrates than farmlands that stimulate the synthesis of soil enzymes [37,38], such as urease, phosphatase, saccharase, cellulase, and catalase activities (*p* < 0.05) (Table 2). In addition, farmland has suffered serious soil erosion in the study area of the Loess Plateau, which has caused severe nutrient loss and has ultimately resulted in lower soil enzyme activities [5,24].

Long-term natural grassland had a lower urease activity (*p* < 0.05) than sloping farmland. Soil microorganisms do not need to secrete more enzymes to obtain additional nutrients because the efficiency of the enzymes is negatively correlated with nutrient availability [39]. To obtain access to more N, soil microorganisms secrete urease when soil N availability is low [6]. The N content in the soil increased significantly through long-term grassland restoration due to the continuous inputs of plants, which provide sufficient N for the growth and metabolism of microorganisms [6]. Moreover, the amylase and polyphenol oxidase activities were also reduced by vegetation restoration compared to sloping farmland (*p* < 0.05) (Table 2), indicating that there was a lower SOM decomposition rate at the late stage of vegetation restoration (~30 years). This can be concluded from the microbial respiratory quotient (qCO_2_) being reduced after farmland was converted to grasslands, shrubs, forests and mixed forests (Figure 4).

Compared with sloping farmland (*p* < 0.05), vegetation restoration increased microbial biomass, which resulted in higher BR in the grasslands, shrubs, forests and mixed forests (Figure 4). This was possibly due to the greater plant diversity, biomass and residues after vegetation restoration providing more nutrient pools and niches for soil microorganisms. The mixed forest had the highest MBC, MBN and MBP (Figure 4), which also indicated that mixed forests are a good measure to improve the soil quality on the Loess Plateau. In addition, the total C, N, and P contents in microorganisms and soil synchronously increased after farmland abandonment, demonstrating that there was a potentially strong interaction between soil and microorganisms following vegetation restoration.

### 4.3. Factor Effects on SOC since Vegetation Restoration

Soil physical-chemical properties have been extensively used to evaluate SOC, however, these properties usually change slowly, and thoroughly reflecting soil changes using these properties is impossible [13]. Soil microbial properties rapidly respond to soil changes caused by both natural and anthropogenic factors, and some enzymes are closely related to soil energy flow and nutrient cycles [13]. For instance, soil microbial biomass is considered to be a transformation agent of soil organic matter (SOM) and a labile pool for plant nutrients [40]. Soil quality indicators have been developed because of the complex nature of soils and the exceptionally large number of soil properties that must be determined. Selection of indicators that appropriately reflect the overall change in soil quality is important.

Soil N dynamics are a key parameter in the regulation of long-term terrestrial C sequestration [41]. This study also found that SOC had significant correlations with AN, TN, AP and TP (Figure 2 and Figure 3). In fact, SOC was closely coupled with TN [9], and SOC showed the same dynamics as soil TP during vegetation restoration [5,6]. In addition, SOC was significantly positively correlated with C/N (*p* < 0.05). Deng et al. [42] reported that SOC was significantly positively correlated with the soil TN and C/N ratio following vegetation restoration. The study also found that SOC was significantly positively correlated with LOC (*p* < 0.01) and NLOC (*p* < 0.01) (Table 3) because LOC and NLSOC are two components of SOC. NLOC is a relatively stable form of soil carbon, and LOC is mainly input into soils by higher plants, which will increase the SOC content even though priming accelerates the decomposition of native SOC [43].

Soil enzyme activities were significantly correlated with the SOC content [15,44], because the transformations of important organic elements are facilitated by microorganisms [45]. However, one study reported polyphenol oxidase was closely related to soil humus decomposition and was not significantly correlated with SOC [13]. This may be related to the different components of the litter and the pathway of humus decomposition in the soils of different species [13]. SOC also had a positive correlation with MBC [46,47]. MBC reflects the size of microbial populations and includes both metabolically active and resting-state microorganisms [6], whereas parameters such as BR reflect the actual and potential microbial activities in the soil [13]. Indeed, soils with more SOC also had higher BR [48], because a large part of the SOC is dedicated to sustaining microbial respiration.

## 5. Conclusions

Land-use change after farmland conversion can increase soil C accumulation. However, the effects of land conversions from farmlands on soil C were varied among grasslands, shrubs, forests and mixed forests. Herein, the mixed forests had the largest positive effects on SOC, followed by the natural grasslands. Vegetation restoration also increased soil N and P content and soil microbial and enzyme activities. Although vegetation restoration increased the basal respiration (BR) of soil microbes, the microbial respiratory quotient (qCO_2_) decreased after farmland conversion. Soil nutrients, such as N and P, and soil microbial activities were the main factors that affected SOC after vegetation restoration. The results suggested that mixed forests such as trees and shrubs (*R. pseudoacacia* and *C. korshinskii*) are the best choice for vegetation restoration after farmland conversion in the central Loess Plateau.

## Figures and Tables

**Table 1:** Information of geographical characteristics and soil physical properties in the five land use types. Note: SF, Sloping farmland; GL, Grassland; SL, shrubland of *Caragana korshinskii*; FL, forestlands of *Robinia pseudoacacia*; ML, mixed forests of *Robinia pseudoacacia* + *Caragana korshinski*. pH, soil pH; BD, soil bulk density. Different lower-case letters mean significant differences in different land use types at 0.05level (*p* < 0.05). Data of soil physical properties are Means ± SE. *n* = 3. All plots of the five land use types were located in the hill-slope, loess-derived soil and north-faced slope.

Land Use Types	Slope (°)	Altitude (m)	pH	BD (g cm^−3^)	Soil Fractions (%)			Primary Undergrowth Vegetations
					Sand (>0.2 mm)	Silt (0.2–0.002 mm)	Clay (<0.002 mm)	
SF (Farmlands)	20–22	1165–1178	8.7 ± 0.2 a	1.27 ± 0.12 a	82.1 ± 0.2	14.8 ± 0.1 c	3.1 ± 0.1 c	*Setaria italica*
GL (Grasslands)	20–23	1189–1202	8.7 ± 0.1 a	1.18 ± 0.12 bc	81.1 ± 0.4	15.9 ± 0.3 b	3.0 ± 0.2 c	*Artemisia sacrorum*
SL (Shrublands)	24–27	1039–1089	8.7 ± 0.1 a	1.19 ± 0.12 b	82.1 ± 0.3	14.6 ± 0.5 c	3.3 ± 0.2 c	*Artemisia sacrorum*, *Stipa bungeana*
FL (Forestlands)	22–25	1119–1234	8.7 ± 0.1 a	1.08 ± 0.12 d	78.6 ± 0.3	17.7 ± 0.4 a	3.7 ± 0.1 a	*Lespedeza bicolor*, *Stipa bungeana*
MF (Mixed forests)	23–27	1087–1165	8.6 ± 0.1 b	1.15 ± 0.12 c	80.8 ± 0.3	16.0 ± 0.2 b	3.2 ± 0.1 b	*Artemisia sacrorum*

**Table 2:** Soil enzymes activities in different vegetation restoration types. Note: SF, Sloping farmland; GL, Grassland; SL, shrubland of *Caragana korshinskii*; FL, forestlands of *Robinia pseudoacacia*;; ML, mixed forests of *Robinia pseudoacacia* + *Caragana korshinski*. Different lower-case letters mean significant differences in different land use types at 0.01 level (*p* < 0.01). Data are Means ± SE. *n* = 3.

Land Use Types	Saccharase (mg Glucose g^−1^ h^−1^)	Cellulase (mg Glucose g^−1^ h^−1^)	Urease (mg NH_4_^+^-N g^−1^ h^−1^)	Amylase (mg Maltcose g^−1^ h^−1^)	Phosphatase (mg Phenol g^−1^ h^−1^)	Polyphenol Oxidase (mL 0.01 N I_2_ g^−1^)	Catalase (mL 0.1 N KMnO_4_ g^−1^)
SF	1.05 ± 0.09 c	1.44 ± 0.19 b	0.57 ± 0.14 d	1.23 ± 0.12 ab	0.32 ± 0.13 c	2.81 ± 0.19 a	0.49 ± 0.17 d
GL	2.49 ± 0.18 b	2.13 ± 0.19 a	0.40 ± 0.12 e	1.01 ± 0.12 bc	1.29 ± 0.22 b	2.16 ± 0.19 b	0.64 ± 0.18 c
SL	2.17 ± 0.27 b	1.95 ± 0.19 a	1.77 ± 0.21 a	0.90 ± 0.23 c	1.32 ± 0.27 b	2.11 ± 0.23 b	0.69 ± 0.09 c
FL	3.27 ± 0.34 a	1.97 ± 0.18 a	1.27 ± 0.11 b	0.85 ± 0.22 c	1.18 ± 0.21 b	2.12 ± 0.20 b	0.96 ± 0.21 a
ML	2.54 ± 0.24 b	1.95 ± 0.26 a	0.60 ± 0.17 d	1.05 ± 0.18 bc	1.56 ± 0.27 a	1.98 ± 0.27 b	0.80 ± 0.19 ab

**Table 3:** Pearson correlation coefficient between soil organic carbon and other soil properties. Note: * Correlation is significant at the 0.05 level (*p* < 0.05) (2 tailed) and ** Correlation is significant at the 0.01 level (*p* < 0.01) (2 tailed). N = 15. ^##^ indicates the value was the explain rates based on the coefficient of determination (*R*^2^) of the multivariable linear regression analysis; ^#^ indicate the residual contribution rate of the multivariable linear regression, which indicates other factor’s contribution to SOC that were not determined in this study.

Factors	Soil Properties	Pearson Correlation Coefficient	Relative Contribution Rate (%)	*p*
Total			81.6 ^##^	
Error			18.4 ^#^	
C fractions	LOC	0.976 **	6.0	<0.01
Soil nutrients	TN	0.937 **	5.8	<0.01
	AN	0.969 **	6.0	<0.01
	TP	0.833 **	5.2	<0.01
	AP	0.818 **	5.1	<0.01
Soil physical properties	pH	−0.477	4.2	>0.05
	BD	−0.097	0.6	>0.05
	Sand	0.039	0.2	>0.05
	Silt	0.370	2.3	>0.05
	Clay	0.180	1.1	>0.05
Soil microbial activities	Urease	−0.089	0.6	>0.05
	Amylase	−0.213	1.3	>0.05
	Phosphatase	0.916 **	5.7	<0.01
	Saccharase	0.615 *	3.8	<0.05
	Cellulase	0.693 **	4.3	<0.01
	Polyphenol oxidase	−0.872 **	5.4	<0.01
	Catalase	0.587 *	3.6	<0.05
	MBC	0.841 **	5.2	<0.01
	MBN	0.353	2.2	>0.05
	MBP	0.813 **	5.0	<0.01
	BR	0.894 **	5.5	<0.01
	qCO_2_	−0.411	2.5	>0.05

## Data Availability

The data presented in this study are available on request from the corresponding author.
